# Accurate anatomic repair of obstetric anal sphincter damage or rectovaginal fistula aided by prior ultrasonograghy: a cohort study

**DOI:** 10.1097/MS9.0000000000000614

**Published:** 2023-04-25

**Authors:** Taole Mokoena, Zeelha Abdool

**Affiliations:** a Department of Surgery; b Department of Obstetrics and Gynaecology, University of Pretoria and Steve Biko Academic Hospital, Pretoria

**Keywords:** anal sphincter, anorectal ultrasonography, case series, layered anatomical repair, obstetric injury, rectovaginal fistula

## Abstract

Anorectal obstetric injuries resulting in anal sphincter damage (ASD) and rectovaginal fistula (RVF) remain a major problem. The resulting flatus or faecal incontinence is devastating. Surgical repair remains a challenge. Postpartum RVF primarily results from ischaemic pressure necrosis following obstructed labour. The fistula tract is surrounded by a fibrous scar. ASD usually results from precipitous labour. The injury heals by fibrous scar leading to varying degrees of anal incontinence. Contraction and retraction of muscles around the injury renders the defect and fibrous scar larger than the primary injury. Anorectal ultrasonography has been used to define RVF and ASD, and the associated fibrous scar.

**Patients and methods:**

A retrospective review of patients who underwent transvaginal surgical repair of RVF and ASD was undertaken. Patients were preoperatively assessed for pathology and incontinence degree. Anorectal ultrasonography was used to define ASD or RVF and the associated scar preoperatively. Repair of RVF or ASD entails total excision of the scar with accurate anatomical layers reconstruction of healthy tissues.

**Results:**

There were 23 patients, 8 RVF with a mean (SD) age 29 (6.78) years and 17 ASD with a mean (SD) age 35.25 (15.90). Twenty followed obstetric trauma (6RVF, 14 ASD), 1 prior rectocoele repair (ASD), 2 rape (1RVF + 1 ASD) and 1 was idiopathic (RVF). All patients had 1 or more prior repairs except for idiopathic RVF. Operative technique entailed transvaginal complete excision of the fibrous scar and accurate anatomical reconstruction of healthy tissue layers. A colostomy was not routinely used. There were three significant postoperative complications: ASD breakdown from an infected haematoma; perianal abscess, later a sinus after drainage; and RVF repair dehiscence during early coitus. All patients had full continence after 8 months minimum follow-up.

**Conclusion:**

Complete excision of the fibrous scar and accurate anatomical tissue layers reconstruction of the obstetric RVF or ASD, aided by prior ultrasonography, yielded good results.

## Introduction

Highlights

- Transvaginal repair of obstetric anal sphincter damage and rectovaginal fistula.
- Prior anorectal ultrasonography defines the extent of damage and the fibrous scar.
- Repair entails fibrous scar excision and accurate anatomic layers reconstruction.
- No routine use of diverting colostomy.
- Good functional results were achieved.

Anorectal obstetric injuries resulting in anal sphincter damage (ASD) and rectovaginal fistula (RVF) remain a major problem^1,2^. The resulting flatus or faecal incontinence is devastating for patients who suffer physical and psychosocial complications. Surgical repair remains a challenge and a plethora of surgical approaches attests to this.

The pathology of postpartum RVF is primarily a result of ischaemic pressure necrosis from obstructed labour^2^. The resulting fistula tract is surrounded by fibrous scar tissue. ASD from third or fourth degree perineal tears, which may be associated with RVF, is usually caused by precipitous second stage of labour, but may also result from instrument delivery or poorly executed episiotomy and their poor primary repair^1,3^. The resulting injury heals by fibrous scar leading to varying degrees of anal incontinence. Contraction and retraction of muscles around the injury renders the defect and resulting fibrous scar larger than the primary injury^4^ analogous to the healing of midline laparotomy wound dehiscence^5^.

A transvaginal repair technique of ASD and RVF was developed and is reported herein. Repair follows established surgical principles of layered anatomical repair of ventral incisional hernia where the scar tissue is totally excised and healthy normal tissues are reconstructed accurately in their anatomical layers^5^. Anorectal ultrasonography is used to define ASD or RVF and their associated scar tissue.

Endoluminal anorectal ultrasound (EAUS) has been shown to accurately delineate anorectal damage differentiating fibrous scar from viable muscle^6^ even in asymptomatic women^7^, and this fibrous scar has been confirmed histologically^8^. Recently, the less intrusive transperineal ultrasonography (TPU) has been favourably compared to EAUS^9^. Transperineal ultrasound tomography further enhances the definition of these injuries.

## Aim

The aim was to review the results of the secondary repair of RVF and ASD.

## Study design

This is a retrospective, single-centre, consecutive case series.

## Setting

University referral teaching hospital setting.

## Patients and methods

A retrospective review of patients managed at the Department of Surgery between 2008 and 2021 was undertaken. Patients were preoperatively assessed for anorectal pathology and degree of incontinence utilising the Wexner scoring^10^. EAUS, and later TPU or transperineal ultrasound tomography, was used to define the injuries. Repair of RVF or ASD entails total excision of associated scar tissue with accurate reconstruction of healthy normal tissues in their anatomical layers.

The study was approved by the Faculty of Health Sciences Research Ethics Committee (reference 492/2015), and was conducted according to the Helsinki Declaration on research on human subjects and reported in line with STR0CSS 2021 Criteria^11^, Supplemental Digital Content 1, http://links.lww.com/MS9/A83. Selected patients gave informed written consent for clinical video photography and scientific publication of the procedure. The research was retrospectively registered on the National Health Research Database

## Surgical technique

A phosphate enema and appropriate intravenous antimicrobial prophylaxis are administered on the morning of the operation. The operation is carried out under general or spinal block anaesthesia. The patient is placed in lithotomy with the buttocks elevated on a sacral cushion and suture-parted or taped away from the anus. The anorectum and vagina are cleaned with povidone-iodine (betadine) and the patient is draped in the usual fashion.

*A key to success of the repair is total excision of the fibrous scar and accurate reconstruction of each individual constitutive anatomical layer whose knowledge is crucial* ^12,13^.

A vertical surgical approach in the urogenital triangle through the vagina is utilised.

No vasoconstricting agents such as adrenalin are used.

Pathology is assessed digitally to confirm prior EAUS findings. A betadine-soaked ribbon gauze or throat pack is inserted deep into the anorectum to protect the posterior rectal wall from injury during surgery.

**Figure 1:**
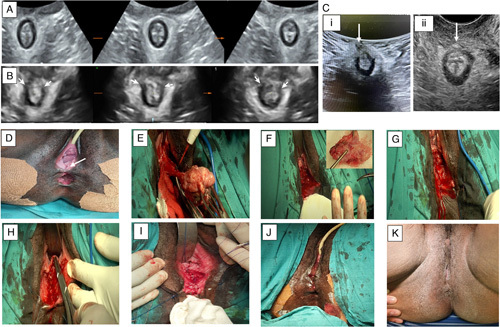
Shows transperineal ultrasound tomography (TUT) of anal sphincter damage (ASD) before and after repair together with selected operative repair sequences of ASD associated with a low rectovaginal (anovaginal) fistula: Panel A illustrates a TUT of normal anal sphincter in nulliparous woman. Panel B shows a TUT of obstetric ASD (between the arrows). Panel C shows TUT and TPU of a healed ASD repair, Ci is 4D TUT at 8 months postoperative, Cii is 2D TPU at 10 months (arrow indicates small residual repair ‘scar’). (D) shows a deformed damaged external anal sphincter and perineum with associated anovaginal fistula opening-arrow. (E) shows the excision of the external anal sphincter muscle scar and associated fistula being completed. (F) shows the resultant ‘cloaca-like’ defect after excision of the anal sphincter scar and fistula (insert). G, H, I and J show selected stages in the layered anatomical repair sequence where, (G) = completed rectal mucosa apposition (anorectal tube reconstruction), (H) = the start of vaginal wall muscle apposition, (I) shows superficial external anal sphincter and superficial transverse perineal muscles apposition. (J) = freshly completed anal sphincter and perineal body reconstruction. (K) shows a healed repair.

Accurate anatomical ASD repair starts with the placement of three stay sutures, one cephalad, two laterally at the prior EAUS estimated extent of the fibrous scar (Fig. 1B). The thus delineated triangular scar tissue is completely excised and healthy normal tissue exposed (Fig. 1E and F). A tension free accurate anatomic tissue layers repair is undertaken. The repair proceeds with the apposition of anorectal mucosa carefully incorporating the submucosa with an interrupted 3/0 polydioxanone (PDS) suture, which is used throughout. Sutures are loosely tied anteriorly within the wound, away from the faecal stream, reconstructing the anorectal tube (Fig. 1G). The rectal wall, internal anal sphincter and deep external anal sphincter muscles layers are individually apposed in turn. The puborectalis, pubovaginalis, deep transverse perineal and vaginal wall muscles, are all individually sutured (Fig. 1H). The superficial external anal sphincter and superficial transverse perineal muscles are apposed next. This automatically reconstructs the perineal body (Fig. 1I). The subcutaneous external anal sphincter and bulbospongiosus muscles are next sutured with inverting interrupted PDS with knots buried in the wound (Fig. 1J). The vaginal mucosa and perineal skin should be adequately apposed, and may only need a few inverting interrupted sutures. However, it is better to leave these open to allow free drainage. The same principle is used for repair of ‘cloaca’ type ASD defects where free fibrous edges are first excised to expose healthy tissue layers, which are individually apposed.

The repair of RVF follows a similar principle: four stay sutures are placed at the proximal, distal and two lateral extents of the fibrous scar. The thus outlined rhomboid or ellipse fistula and surrounding scar tissue are excised *en bloc*. Rectal mucosa, rectal and vaginal wall muscles are sequentially sutured individually with interrupted PDS. The vaginal mucosa should be automatically apposed and requires no suturing.

Low anovaginal fistulae with invariably scarred external anal sphincter muscles are managed as for ASD after excision of the fistula and sphincter muscle scar (Fig. 1D and E) , Supplemental Digital Content 2, http://links.lww.com/MS9/A84, Supplemental Digital Content 3, http://links.lww.com/MS9/A85.

No covering colostomy is used except during the early evolution of the repair technique. However, if the patient came in with a prior colostomy performed elsewhere, this was left *in-situ* and colostomy is reversed after at least 6 weeks.

Antiseptic (acriflavine) soaked gauze plugs are left in the vagina and anorectum overnight as well as a urinary catheter.

A stool softener, Psyllium, and a lubricant liquid paraffin, are prescribed twice daily for 10–14 days, as well as frequent povidone-iodine sitz baths and vaginal douches especially after a bowel motion. Anusol suppository and anethaine (tetracaine) cream are recommended before bowel movement. Patients are allowed free diet and restricted from coitus for 3 months.

## Statistics

Nominal comparison and proportions were expressed in percentages. No specific statistical operation was undertaken.

## Results

There were 23 patients, 8 had RVF and 17 had ASD where 2 had both RVF and ASD (Table 1). The majority (87%) followed obstetric trauma. A majority (65.22%) had 1 or more prior repairs elsewhere in addition to primary repair of episiotomy or primary obstetric perineal laceration.

**Table 1:** Findings in 23 female patients with rectovaginal fistulae or anal sphincter damage

	RVF (*n*=8)^*^	ASD (*n*=17)
Age (years)		
mean (SD)	29 (6.98)	35.25 (15.90)
range	19–36	20–65
Mechanism of Injury		
-obstetric trauma	6	14
-rape	1	1
-previous rectocele repair	–	1
-idiopathic	1	–
-unrecorded	–	1
Associated pathology		
-anovaginal fistula	2	2
-cloacal defect	–	4
Degree of Incontinence		
-solid stool	Nil	4
-liquid stool	8	17
-flatus	8	17
Previous Repair^†^		
-x1	4	3
-≥2	3	5
Preoperative Ultrasonography	4	7

* Some patients (2) had both RVF and ASD.

† Characterisation of previous repair excludes primary repair of episiotomy or primary obstetric perineal laceration.

ASD, Anal Sphincter Damage; RVF, Rectovaginal Fistula.

There were three significant postoperative complications (Table 2):

- An infected haematoma lead to the breakdown of the ASD repair, which was successfully redone with a covering colostomy.
- A perianal abscess, which was surgically drained and later developed into a perianal sinus. This was excised and the ASD repair remained intact,
- RVF repair dehiscence during early coitus was successfully redone with a covering colostomy, which was reversed in 3 months.

**Table 2:** Outcome of surgical repair in 23 patients with rectovaginal fistula or anal sphincter damage

	RVF	ASD
Preoperative		
Colostomy^*^	3	5
Complications^†^		
-haematoma	1	1
-wound sepsis	1	2
-perianal abscess	1	1
-perianal sinus	–	1
Repair dehiscence		
-infected haematoma	–	1
-early coitus	1	–
-subsequent vaginal delivery	–	1
Continence at follow-up 6 – 12 mo	Good	Good

* Some patients presented with colostomy from elsewhere.

† Some patients had more than one complication for example, patient with perianal abscess leading to perianal sinus.

ASD, anal sphincter damage; RVF, rectovaginal fistula.

During the early evolution of this technique, covering colostomy was routinely used. These patients had a retention barium enema, anorectal manometry and EAUS performed to confirm the integrity of the repair. Some of these ASD repair patients needed anal dilatation for anal stenosis before colostomy reversal. No patients needed anal dilatation since the use of a colostomy was dispensed with.

All patients showed good continence on 8 months minimum follow-up. In some patients, anorectal ultrasonography was performed to confirm the integrity of the repair after resumption of coitus (Fig. 1C). Three patients are known to have had subsequent vaginal deliveries and 1 sustained a breakdown of the repair resulting in a ‘cloaca-type’ ASD defect. This was successfully repaired.

A similar surgical approach was adopted for cases of traumatic posterior damage of the anorectal anatomy complex with resultant incontinence or posterior anorectal cutaneous fistulae. EAUS was used to define the anatomical defect and extent of fibrous scar. The operative procedure is similar except that the patient is placed prone in a jack-knife position.

## Discussion

Delayed or repeat repair of anorectal anatomy disruption with resultant RVF or ASD is a major challenge. Many surgical approaches have been described. Many incorporate the unhealthy fibrous scar in the repair, for example, overlap repair^4,14^, endorectal, transvaginal or transperineal mucosal advancement flap^15,16^ with muscle plication^17^, labial fat pad^15,16^ or internal anal sphincter muscle^16^ transfer over the RVF defect and a transvaginal purse–string repair^18^.

A surgical repair technique was developed that entails complete excision of ultrasonographically or digitally defined fistula and fibrous scar tissue, and accurate anatomical suture apposition of healthy mucosa and each individual muscle layers by a transvaginal approach. Adelawo *et al.* described a similar transvaginal nonoverlapping layered RVF repair after total excision of the fistula tract using a disposable biopsy punch^19^. Fu J *et al.* described a transanal layered repair of RVF following complete excision of the fistula to guarantee a margin of healthy tissue and stated that interposition of labial fat or muscle did not improve their cure rate^20^. The latter two publications stress the complete excision of the fistula and the scar, and the use of healthy tissue for a layered repair, which we ascribe to. The use of biological mesh repair of RVF has yielded variable success rates by different authors^21^.

Wiskind and Thompson reported a transverse transperineal multilayered repair of low RVF encompassing puborectalis, bulbocavernosus, transverse perineal and external anal sphincters muscles repair^22^. Chew and Reiger used EAUS to delineate and excise the RVF scar for their transperineal overlapping anal sphincteroplasty^23^. The Parks repair of ASD excises the bulk of the scar and reconstructs the anorectal mucosa tube but the fibromuscular layer is repaired by the overlap technique^4^. Its results deteriorated with time^14^. Our approach for secondary repair of ASD entails complete excision of the fibrous scar and an accurate individual anatomic layers apposition. Hibbard reported a technique where ASD or perineal body lacerations were repaired in layers including pubococcygeal muscle^24^. The use of an artificial anal sphincter or muscle interposition for anal incontinence is complicated and requires much experience. It is probably suited for patients with neurological deficit as well as ASD^25^.

A caesarean section is advised for subsequent deliveries to avoid disruption of the repair.

Our technique has given very rewarding results.

A limitation of the study includes its retrospective nature and lack of long-term follow-up. A prospective controlled study, preferably multicentre to recruit enough patient with extended follow-up would be necessary to confirm these results.

## Conclusion

Complete excision of the fibrous scar and accurate anatomical tissue layers reconstruction of secondary repair of obstetric RVF or ASD, aided by prior anorectal ultrasonography, yielded good results.

## Ethical approval

The study was approved by Faculty of Health Sciences Research Ethics Committee (reference 492/2015).

## Consent

NA.

## Sources of funding

The study was funded from internal Department of Surgery financial resources.

## Author contributions

T.M.: was the surgeon, he conceived and designed the study, he collected and analysed the data, and drafted the manuscript. Z.A.: performed and analysed transperineal ultrasonographs, and she edited the draft manuscript.

## Conflicts of interest disclosure

The authors declare no conflict of interests.

## Research registration unique identifying number (UIN)

1. Name of the registry: National Health Research Database.
2. Unique Identifying number or registration ID: GP_202209_012.
3. Hyperlink to your specific registration (must be publicly accessible and will be checked): https://nhrd.health.gov.za/Proposal/SearchResults?UnRef=GP_202209_012

## Guarantor

Taole Mokoena.

## Provenance and peer review

The report is the original work of the principal author. It has not been commissioned or sponsored by any individual or institution, and it was externally peer reviewed.

## Supplementary Material
